# Randomized Trial Assessing the Impact of Routine Assessment of Health-Related Quality of Life in Patients with Head and Neck Cancer

**DOI:** 10.3390/cancers13153826

**Published:** 2021-07-29

**Authors:** Oumar Billa, Franck Bonnetain, Jérôme Chamois, Angeline Ligey, Valérie Ganansia, Georges Noel, Sophie Renard, Sophie Maillard, Magali Quivrin, Noémie Vulquin, Pierre Truntzer, Tienhan Sandrine Dabakuyo-Yonli, Philippe Maingon

**Affiliations:** 1 Georges-François Leclerc Cancer Centre-UNICANCER, Epidemiology and Quality of Life Unit, 21000 Dijon, France; obilla@cgfl.fr; 2 Lipids, Nutrition, Cancer Research Center, U1231 INSERM, 21000 Dijon, France; 3 National Quality of Life and Cancer Clinical Research Platform, 21000 Dijon, France; 4 Methodology and Quality of Life in Oncology Unit, Inserm UMR 1098, University Hospital of Besancon, 25000 Besancon, France; fb@chu-besancon.fr; 5 Georges-François Leclerc Cancer Centre-UNICANCER, Radiotherapy Department, 1 Rue Professeur Marion, 21000 Dijon, France; jchamois@vivalto-sante.com (J.C.); Mquivrin@cgfl.fr (M.Q.); Nvulquin@cgfl.fr (N.V.); Philippe.maingon@aphp.fr (P.M.); 6 Centre Hospitalier Saint Gregoire, 35760 Saint-Grégoire, France; 7 Centre Hospitalier Fleriat, 01012 Bourg-en-Bresse, France; Aligey@ch-bourg01.fr; 8 Paul Strauss Cancer Centre-Unicancer, 67000 Strasbourg, France; Vganansia@strasbourg.unicancer.fr (V.G.); Gnoel@strasbourg.unicancer.fr (G.N.); Ptruntzer@strasbourg.unicancer.fr (P.T.); 9 Institut de cancérologie de Lorraine, 54519 Vandœuvre-lès-Nancy, France; Srenard@nancy.unicancer.fr; 10 Centre Bourgogne, 59000 Lille, France; Smaillard@ch-bourg01.fr; 11 Radiotherapy Unit, Hôpital de la Pitié-Salpêtrière-APHP, 75013 Paris, France

**Keywords:** routine assessment, quality of life, head and neck cancer

## Abstract

**Simple Summary:**

The purpose of this research was to investigate the impact of routine assessment of health-related quality of life (HRQoL) on quality of life and satisfaction with care in patients with head and neck cancer (HNC). A randomized controlled open-label clinical trial with 200 patients with HNC managed in four cancer centers in Eastern France was performed. In the intervention arm (regularly completed HRQoL questionnaires), HRQoL mean change was significantly improved at 2 years from baseline. Compared with the control arm, differences were not statistically significant, but minimal clinically important differences in favor of the intervention arm were found for HRQoL, satisfaction with waiting times, and satisfaction with accessibility. In patients with head and neck cancer undergoing treatments, routine assessment of HRQoL is a simple practice and may have HRQoL and satisfaction benefits.

**Abstract:**

The impact of routine assessment of health-related quality of life (HRQoL) on satisfaction with care and the HRQoL of patients with head and neck cancer (HNC) treated with radiotherapy was assessed. Patients with HNC were randomly assigned to two arms, with stratification on sex, cancer localization, and stage of the disease. In the intervention arm, the patients completed the EORTC QLQ-C30 and EORTC QLQ-H&N35 questionnaires first before randomization, then before each medical appointment during radiotherapy (7 weeks), and then every 3 months until 1 year and at 2 years thereafter. In the control arm, the EORTC QLQ-C30 and EORTC QLQ-H&N35 questionnaires were completed before randomization and at 1 year and 2 years thereafter. The primary endpoint was mean change in HRQoL at score at 2 years from baseline assessed by EQ VAS from the EuroQol questionnaire. The secondary endpoint was mean change in satisfaction with care at 2 years from baseline assessed by QLQ-SAT32. Two hundred patients with head and neck cancers were involved in this study (mean age, 58.83 years (range, 36.56–87.89)), of whom 100 were assigned to the intervention arm and 100 to the control arm. Patients in the intervention arm were reported to have a statistically significant increase in EQ VAS at 2 years (*p* < 0.0001) and exceeded the minimal clinically important difference (mean change at 2 years from baseline = 10.46). In the two arms, mean differences between arms were not statistically significant, but minimal clinically important differences in favor of the intervention arm were found for EQ VAS (mean change difference (MD) = 5.84), satisfaction with care, in particular waiting times (MD = 10.85) and satisfaction with accessibility (MD = 6.52). Routine assessment of HRQoL improves HRQoL and satisfaction with care for patients with HNC treated with radiotherapy.

## 1. Introduction

Head and neck cancers (HNC) represent 4% of all cancers worldwide. The main risk factors for HNC are tobacco smoking, alcohol consumption, and HPV infection [1,2,3,4]. For nonmetastatic HNC, the reference treatments remain to be surgery and radiotherapy. In addition to the negative effects of disease, among patients treated with radiotherapy, the treatment itself can induce debilitating side effects, such as skin reactions, dysphagia, mucositis, anorexia, and xerostomia, with discomfort and pain [5,6,7,8,9,10]. These effects not only affect patients physically but also can lead to psychosocial problems, thus negatively influencing health-related quality of life (HRQoL) in this population [11,12,13,14,15,16].

Nowadays, with the ever-increasing numbers of cancer survivors, special attention to HRQoL is warranted among patients in oncology. Accordingly, HRQoL assessment has become a key endpoint in cancer management and clinical trials. HRQoL has the advantage of taking account of the patient’s perception of his or her disease and treatment, providing additional insights beyond the clinical information. Furthermore, the use of HRQoL data in clinical practice has been shown to help guide the choice of treatment [17], facilitate the detection of toxicities [18,19,20], enable daily monitoring, provide useful information to physicians, facilitate communication, and assist physicians in decision making, and it may also increase survival [21,22,23]. However, the impact of routine assessment of HRQoL on the patient’s well-being, quality of life, and satisfaction has not been widely investigated in the setting of HNC [10,21,24].

We conducted a phase III randomized multicenter study to assess the impact of routine assessment of HRQoL on HRQoL and satisfaction with care in patients with HNC.

## 2. Materials and Methods

### 2.1. Study Design

In this prospective multicenter phase III study, patients with HNC were randomized 1:1 into 2 arms. In the intervention arm, patients were invited to complete the EORTC QLQ-C30 and EORTC QLQ-H&N35 [25,26,27] questionnaires first before randomization, then before each medical appointment during radiotherapy (7 weeks), then every 3 months until 1 year and at 2 years thereafter. In the control arm, the EORTC QLQ-C30 and EORTC QLQ-H&N35 questionnaires were also completed before randomization and at 1 year and 2 years thereafter.

The trial complied with the Helsinki Declaration and Good Clinical Practice guideline and was approved by the EST I ethics committee and the Agence Francaise de Securite sanitaire des produits de santé. The trial was registered on the clinical Trial.gov website (NCT 01210872).

### 2.2. Participants

Patients were recruited in 4 cancer centers in Eastern France: George-François Leclerc Center, Paul Strauss Center, Jean Godinot Institute, and Lorraine Cancerology Institute. The eligibility criteria were: (1) diagnosis of primary nonmetastatic HNC, (2) treatment with radiotherapy delivered by intensity-modulated radiation therapy as the initial treatment, (3) age of over 18 years, (4) ability to read and speak French, and (5) provision of written informed consent to participate. Patients with a history of other cancer, patients with a second primary cancer at the time of diagnosis, and patients with a history of psychiatric disorders were excluded.

### 2.3. Randomization

Randomization was performed using TENALEA (Trans European Network for Clinical Trials Services) software. Patients were randomly assigned at a 1:1 ratio to 1 of the 2 arms of the study according to a minimization method and with stratification on sex, cancer localization, stage, and center. The Biostatistics Unit of the Georges Francois Leclerc Center generated the random allocation sequence and sent the randomization number and patient assignation to intervention to the study investigators via e-mail. The participants, investigators, and healthcare team could not be blinded to the allocation sequence.

### 2.4. Study Procedure

At the first medical appointment, eligible patients were informed about the study by their oncologist. If interested in participating, they were referred to the research team, who explained the study procedures. After the participants received explanations and provided informed consent, they completed the baseline self-report questionnaires before being randomly allocated to either the intervention or the control arm. The patients’ sociodemographic characteristics, such as age, sex, smoking status, marital status, body mass index (kg/m^2^), and clinical data, such as cancer subsite, cancer stage, comorbidities as defined by the Charlson comorbidity index (categorized in 2 groups, i.e., no comorbidity and at least 1 comorbidity), current alcohol consumption, and treatments, were collected. Follow-up self-report questionnaires were given to the participants by a clinical research technician before each medical appointment with physicians, either in the waiting room or in the patient bedside, or in some cases, the questionnaires were posted to the participants.

### 2.5. Intervention

The intervention in this study was routine assessment of HRQoL using validated HRQoL questionnaires. The used intervention questionnaires were the EORTC QLQ-C30 questionnaire [25] and the EORTC QLQ-H&N35 specific module for HNC [26]. Both arms completed the EORTC QLQ-C30 and EORTC QLQ-H&N35 questionnaires at baseline before randomization and prior to radiotherapy, but HRQoL assessment times during follow-up were different between arms.

In the intervention arm, the intervention (i.e., routine assessment of HRQoL) consisted of regular completion of self-report questionnaires at each medical appointment throughout the follow-up period, until the end of the study at 2 years. Accordingly, the patients in the intervention arm regularly completed the intervention questionnaires every week during radiotherapy (which lasted 7 weeks), then every 3 months up to 1 year after radiotherapy, and then 2 years after radiotherapy. A duplicate of their self-report questionnaires, with the corresponding HRQoL scores generated, was made available to the physician prior to the corresponding medical appointment.

In the control arm, no HRQoL assessments were performed until 1 year and then at 2 years after radiotherapy (Table 1). No feedback regarding the HRQoL scores was given to the physician.

The EORTC QLQ-C30 questionnaire measures common cancer-related symptoms and is composed of 30 items that generate 15 scales, namely, 5 functional scales (physical, role, cognitive, emotional, and social), 8 symptom scales (fatigue, nausea and vomiting, pain, dyspnea, insomnia, appetite loss, constipation, and diarrhea), global health status, and financial difficulties [25]. The EORTC QLQ-H&N35 comprises 35 questions assessing symptoms and side effects of treatment, social function, and body image, and contains 7 multi-item symptom scales (pain, swallowing, senses (taste and smell), speech problems, trouble with social eating, trouble with social contact, and less sexuality), 6 single-item symptoms (problems with teeth, problems with opening mouth, dry mouth, sticky saliva, coughing, and feeling ill), and 5 additional items related to the use of painkillers, nutritional supplements, feeding tube, weight loss, and weight gain [26]. HRQoL scores vary from 0 (worst) to 100 (best) for the functional and global health scales and from 0 (best) to 100 (worst) for the symptom scales.

### 2.6. Trial Endpoints

The primary endpoint was mean change in HRQoL score at 2 years from baseline. The EuroQol questionnaire (EQ-5D) was used to assess the mean change in health global state (EQ VAS) at 2 years from baseline. EQ-5D comprises 2 parts: The first part is the EQ-5D index, which explores 5 dimensions (mobility, self-care, usual activities, pain/discomfort, and anxiety/depression), and each dimension has 3 levels: no problems, some problems, and severe problems. It can be presented as a global health index and produces a composite score between 0 and 1. The second part is a visual analog scale (EQ VAS), which assesses global health state with a score of 100 corresponding to the “best imaginable health state” and a score of 0 for the “worst imaginable health state” [28,29].

The secondary endpoint is mean change in satisfaction at 2 years from baseline, as assessed using the EORTC QLQ-SAT32 questionnaire [30]. It contains 3 subscales: satisfaction with doctors (interpersonal qualities, technical skills, information, and availability), satisfaction with nurses (interpersonal qualities, technical skills, information, and availability), satisfaction with services (interpersonal quality/information, waiting times, accessibility, and exchange of information), and the single overall satisfaction item [30]. Scores range from 0 to 100, with a higher score indicating a greater level of satisfaction with care.

### 2.7. Statistical Analyses

Two hundred patients (*n* = 200) were required to detect a difference of 10 points in HRQoL scores between the two arms with a significance level of 0.01 and a power of 80%. Continuous variables were described as mean ± standard deviation (SD), or median and range, and categorical variables were described as number and percentage. Between-arm differences in continuous variables were compared using Wilcoxon–Mann–Whitney tests, and a chi-square or Fisher’s test was used to compare categorical variables between arms. For endpoints, the mean change difference between the 2 arms was compared using Mann–Whitney tests. HRQoL and satisfaction scores at baseline and after 2 years within each arm were compared using Wilcoxon signed-rank tests. A negative change score indicated a decline in HRQoL and satisfaction. Following the guidelines of Osoba et al., the minimal clinically important difference (MCID) was defined in these analyses as a mean difference of least 5 points in the mean change in HRQoL and satisfaction scores [31]. Mixed models for longitudinal HRQoL data (for each selected score with MCID ≥5 points) were used to examine change over time in the differences between arms and the interaction effects. The variables included were study arm, stratification factors, age, current alcohol consumption, comorbidities, and interaction between study arm and time. All tests were two sided, and a *p*-value of 0.01 was considered significant for endpoints. All analyses were performed using SAS, version 9.4 (SAS Institute, Inc., Cary, NA, USA).

## 3. Results

### 3.1. Participant Enrollment and Baseline Characteristics

**Figure 1:**
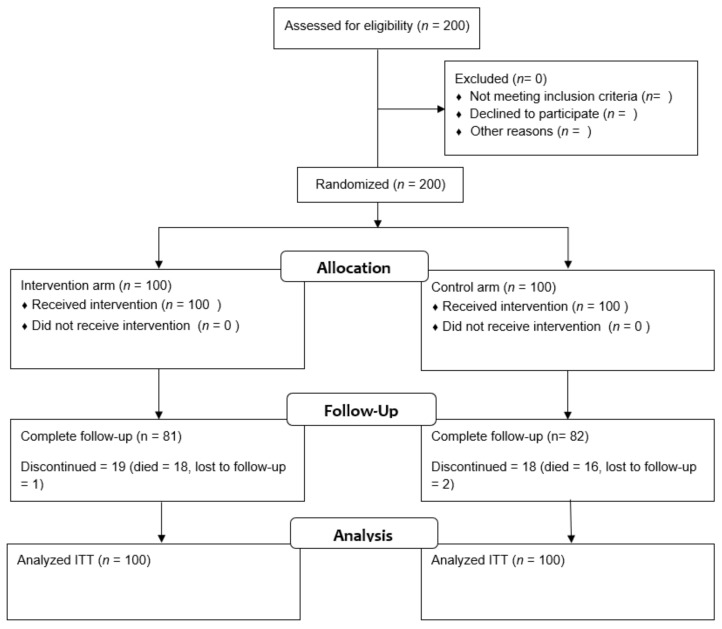
Consort flow diagram of progress through the randomized study. ITT: intention to treat.

From May 2009 to September 2014, 200 patients were enrolled in four cancer centers, of whom 100 were randomly assigned to the intervention arm and 100 to the control arm. During follow-up, 19 patients in the intervention arm and 18 patients in the control arm discontinued the study (Figure 1).

The median age was 58.83 years (range, 36.56–87.89) in the intervention arm and 56.70 years (range, 39.80–86.56) in the control arm. Sociodemographic and clinical characteristics were similar between the two arms, except for comorbidities and current alcohol consumption (Table 2). In the control arm, the patients had more comorbidities (86% vs. 73%, *p* = 0.02) and were less alcohol drunk (18.75% vs. 36.08, *p* = 0.007).

At baseline, there were no statistically significant differences in any dimensions of QLQ-C30 and the QLQ-H&N35 between the two arms (Figure S1A,B in Supplementary Materials).

### 3.2. Intervention Effects on HRQoL and Satisfaction

**Figure 2:**
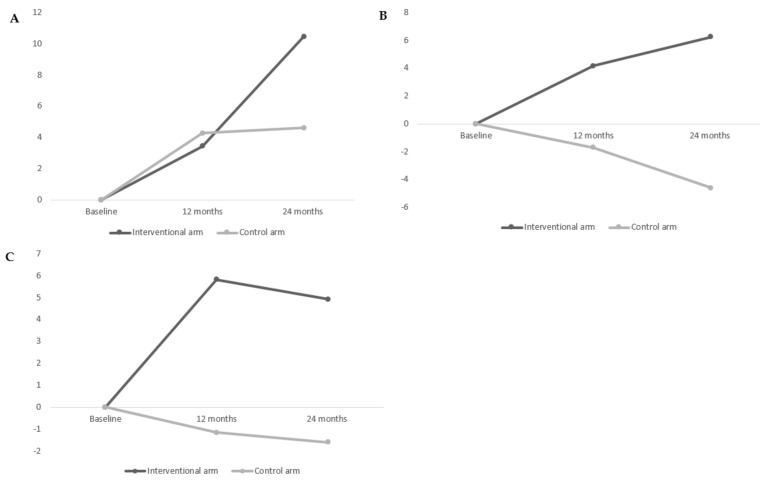
Evolution of difference of scores (with respect to baseline scores), (**A**) EQ VAS, (**B**) satisfaction with waiting times, and (**C**) satisfaction with accessibility between both arms.

The mean EQ VAS scores at baseline were 65.50 (SD = 24.10) and 68.80 (SD = 19.50) in the intervention and control arms, respectively (Table 3). The mean EQ VAS score of the intervention arm had a statistically significant increase of 10.46 between baseline and 2 years (mean change = 10.46, *p* < 0.0001); moreover, this mean change was clinically significant (Figure 2A). In the control arm, there was a mean EQ VAS score increase of 4.62 between baseline and 2 years, but it was not statistically significant (mean change = 4.62, *p* = 0.0698). The comparison of mean change scores between the two arms at 2 years was not statistically significant, but the mean difference (MD) in the mean change at 2 years between the two arms was +5.84 points, which corresponds to a clinically meaningful change (i.e., ≥5 points) in global self-rated health (Table 3, Figure 2A).

Regarding the secondary endpoint, overall satisfaction and all subscales of satisfaction were not statistically significant at any time and between both arms. However, the mean difference at 2 years between the arms in subscale satisfaction with waiting time met the criterion for a clinically meaningful difference (mean increase of 6.25 (SD = 21.81) in the intervention arm vs. mean decrease of 4.60 (SD = 25.07) in the control arm, and mean difference = +10.85) (Table 4, Figure 2B). Similarly, for subscale satisfaction with an accessibility score, the mean difference between the arms was clinically meaningful (mean increase of 4.92 (SD = 26.50) in the intervention arm vs. mean decrease of 1.60 (SD = 24.53) in the control arm, and mean difference = +6.52 points) (Table 4, Figure 2C).

In mixed models adjusted, regardless of the study arm, EQ VAS scores increased up to 2 years (*p* = 0.002) (Supplementary Table S1). The relationship between the study arm and EQ VAS score was not statistically significant (*p* = 0.42). For satisfaction subscales, the study arms were not statistically associated with the patients’ satisfaction with waiting time (*p* = 0.34) and also with the patients’ satisfaction with access to the hospital (*p* = 0.25).

### 3.3. Concomitant Treatments

During the study, painkillers were the most frequently used concomitant drugs with 82.02% in all the patients (Table 5). Use of painkillers was comparable between the arms (78.02% vs. 86.02%, *p* = 0.1552). Additionally, there were no statistically significant differences between the arms concerning the use of concomitant medications, such as antidiarrheal agents, antiemetics, psychotropic drugs, and antibiotics (*p* > 0.05).

## 4. Discussion

This randomized phase III study was conducted in patients with HNC to assess the impact of the routine use of HRQoL measurement on patients’ HRQoL and satisfaction with care. Assessing HRQoL is important for patients with HNC, for whom treatment is burdensome with many potential side effects [32,33]. In this study, the median age was 59.65 years, and males were most frequently affected. Similar results were found in previous studies [14,34], and this could be explained by lifestyle behaviors, such alcohol consumption and tobacco, which are mostly observed in males [34].

In this study, we found that the routine assessment of HRQoL had an impact on the patients’ HRQoL. In the intervention arm, HRQoL scores increased significantly at 2 years from baseline, and this improvement was clinically meaningful. In the control arm, HRQoL score increased, but the increase was neither statistically nor clinically significant. Moreover, a comparison of the mean change between both arms showed a greater mean difference in the intervention arm, with a clinically meaningful improvement of 5.84 points in favor of the intervention. Our findings are in keeping with the studies by Velikova et al. and Basch et al. [20,23], who found an improvement of HRQoL in the intervention arm. Indeed, the routine assessment of HRQoL gives patients the possibility to talk about their needs more frequently, thus improving the patient–physician relationship. Although this was not directly measured in our study, several studies have shown a positive impact of routine assessment of HRQoL on communication [24,35,36]. In the study of Santana et al. [36] with lung transplant patients, they found a positive impact on patient–physician communication but no beneficial effect on the patients’ HRQoL. This finding contrasts with our results but could be explained by the difference in study populations.

Assessment of satisfaction with care, which provides feedback from patients and takes into account their expectations and perceptions, showed a high overall satisfaction score at baseline and at 2 years in the intervention arm. With a mean change difference of 4.5 points between arms (very close to the MCID criterion of ≥5 points), overall satisfaction showed a trend towards a clinically meaningful impact in the intervention arm. Moreover, for two subscales of satisfaction with service, namely, satisfaction with waiting times and magnitude in satisfaction with accessibility, we found a clinically meaningful difference in the intervention arm. This result is novel in that few studies have shown positive effects of routine assessment on patient satisfaction [20,37]. Indeed, Hilarius et al. [35] reported high levels of satisfaction but no significant difference between groups. Additionally, in a recent review [38], four studies assessing satisfaction in routine use of HRQoL in clinical practice showed no significant difference in patient satisfaction. This lack of significant difference could be explained by the fact that patients with cancer generally report a high level of satisfaction, leaving little room for improvement; this phenomenon is termed ceiling effect [23,35,37,38]. Routine assessment of HRQoL in clinical practice may allow patients to feel more engaged as actors in their own health and thus increase their satisfaction [23].

We expected that HRQoL questionnaire completion may motivate patients to discuss more their health issues at their medical appointments, especially in the intervention arm, where duplicates of their HRQoL questionnaires with quality of life score generated were transmitted prior to their medical appointments. Indeed, no significant difference was found in using concomitant treatments, such painkillers, antiemetics, and antibiotics, between the intervention arm and the control arm. Santana et al. [35] reported a significant effect of routine assessment of HRQoL measures on patient management, with a more frequent change in medications in the intervention group. Our study also found that independent of other factors, stage of disease could impact global health state, satisfaction with waiting time, and satisfaction with accessibility. HRQoL and satisfaction scores were highest in patients with early-stage disease. This result again underlines the need for early diagnosis of cancers, with improving patient management.

Our study has some potential limitations. We used a generic measure (EQ-5D) to assess HRQOL as an outcome, rather than a cancer-specific measure, such as the FACT-G [39], which makes it possible to assess several dimensions of HRQoL. This choice is explained by the fact that the EQ-5D questionnaire is widely used in France and easy to understand for patients. Moreover, this choice aimed to avoid confusion with the intervention questionnaires (EORTC QLQ-C30 and EORTC QLQ-H&N35), which are cancer-specific measures.

The strengths of our study include the use of validated tools to assess HRQoL and the randomized trial design, which should increase the robustness of our analysis and limit potential bias in the conclusions. A further strongpoint is the follow-up over a period of 2 years. HRQoL has not been widely studied in clinical trials as a primary outcome measure, and therefore, this study with HNC patients could be a starting point for future research to confirm whether the use of routine HRQoL may influence global health state and satisfaction in these patients.

## 5. Conclusions

The results of the present study show that repeated routine assessment of HRQoL in patients with HNC improves patients’ HRQoL and has a positive impact on some subscales of satisfaction with service. This intervention has the potential to improve clinical practice, and its implementation should be encouraged in routine clinical care with a view to improving a comprehensive patient care.

## Figures and Tables

**Table 1:** Follow-up summary table.

	Baseline (before Randomization and Radiotherapy		Every Week during Radiotherapy (7 Weeks)		At 3, 6, and 9 Months after Radiotherapy		At 1 Year after Radiotherapy (+/−2 Months)		At 2 Years after Radiotherapy (+/−2 Months)	
	Intervention	Control	Intervention	Control	Intervention	Control	Intervention	Control	Intervention	Control
QLQ-C30 ^a^	X	X	X		X		X	X	X	X
H&N35 ^a^	X	X	X		X		X	X	X	X
EUROQOL ^b^	X	X					X	X	X	X
SAT32 ^b^	X	X					X	X	X	X
Clinical variables	X	X	X	X	X	X	X	X	X	X

^a^ intervention questionnaires; ^b^ endpoint questionnaires.

**Table 2:** Baseline sociodemographic and clinical characteristics.

	TOTAL	Intervention	Control	*p*-Value
	N = 200	(N = 100)	(N = 100)	
	*n* (%)	*n* (%)	*n* (%)	
Age median (Min–Max)	59.65 (36.56–87.89)	58.83 (36.56–87.89)	56.7 (39.8–86.56)	0.61
Sex				
Men	154 (77.00)	77 (77.00)	77(77.00)	1.00
Women	46 (23.00)	23 (23.00)	23 (23.00)	
Body mass index (kg/m^2^)	23.97 (20.66–27.55)	25 (20.70–28.15)	23.11 (20.59–26.28)	0.14
Charlson comorbidity score				0.02
At least one	159 (79.50)	73 (73.00)	86 (86.00)	
No comorbidity	41 (20.50)	27 (27.00)	14 (14.00)	
Smoking status				0.61
Nonsmoker	26 (13.20)	12 (12.00)	14 (14,43)	
Current smoker/former smoker	171 (86.80)	88 (88.00)	83 (85.57)	
Missing data	3	-	3	
Current alcohol consumption				0.007
No	140 (70.35)	62 (63.92)	78 (81.25)	
Yes	53 (26.63)	35 (36.08)	18 (18.75)	
Missing data	7	3	4	
Cancer subsite				0.96
Oral cavity	41 (20.60)	20 (20.00)	21 (21.21)	
pharynx	89 (44.72	46 (46.00)	43 (43.43)	
Larynx	35 (17.59)	18 (18.00)	17 (17.17)	
Sinus	13 (6.53)	7 (7.00)	6 (6.06)	
Salivary glands and others	22 (10.55)	9 (9.00)	13 (12.12)	
Cancer stage				0.62
I	55(29.57)	27 (28.42)	28 (30.77)	
II	61(32.80)	35 (36.84)	26 (28.57)	
III	70 (37.63)	33 (34.74)	37 (40.66)	
Surgery				0.48
Yes	180 (90.45)	89 (89.00)	91 (91.92)	
No	19 (9.55)	11 (11.00)	8 (8.08)	
Missing data	1		1	
Chemotherapy				0.79
No	87 (44.39)	43 (43.43)	44 (45.36)	
Yes	109 (55.61)	56 (56.57)	53 (54.64)	
Missing data	4	1	3	

**Table 3:** Health-related quality-of-life endpoint at baseline and 1 and 2 years.

	Baseline Mean Score (SD)			Mean Change from Baseline to 1 Year (SD)			Mean Change from Baseline to 2 Years (SD)			
EuroQol-5D	Intervention (*n* = 100)	Control (*n* = 100)	*p*	Intervention	Control	*p*	Intervention	Control	MD	*p*
EQ-D5 index	0.72 (0.25)	0.76 (0.26)	0.44	0.04 (0.24)	0.04 (0.24)	0.91	0.07 (0.19)	0.06 (0.23)	0.01	0.43
EQ VAS	65.50 (24.10)	68.80 (19.50)	0.63	3.44 (17.78)	4.28 (18.48)	0.33	10.46 (19.00) ^a^	4.62 (21.26)	5.84	0.49

*p*-Value calculated from Mann–Whitney tests; MD: mean difference between intervention and control. ^a^: significant *p*-value from Wilcoxon signed-rank test.

**Table 4:** Satisfaction with care endpoints at baseline and 1 and 2 years.

	Baseline Mean Score (SD)			Mean Change from Baseline to 1 Year (SD)			Mean Change from Baseline to 2 Years (SD)			
QLQ-SAT32 Scales	Intervention (*n* = 100)	Control (*n* = 100)	*p*	Intervention	Control	*p*	Intervention	Control	MD	*p*
Satisfaction with Doctors										
Interpersonal qualities	77.70 (21.20)	74.19 (21.30)	0.26	1.68 (19.51)	−4.08 (26.68)	0.21	1.25 (21.06)	−2.13 (26.42)	3.38	0.62
Technical skills	78.80 (15.90)	75.10 (18.60)	0.27	2.34 (16.87)	0.36 (21.13)	0.57	1.83 (17.03)	−1.01 (20.51)	2.85	0.82
Information	76.20 (19.80)	71.40 (22.00)	0.13	0.66 (20.71)	−3.99 (29.47)	0.18	−2.19 (15.24)	−2.18 (25.84)	−0.005	0.59
Availability	75.50 (20.80)	68.00 (23.70)	0.05	1.12 (18.81)	−1.04 (25.63)	0.74	1.25 (19.77)	1.45 (28.24)	−0.2	0.83
Satisfaction with Nurses										
Interpersonal qualities	80.00 (19.70)	76.65 (21.10)	0.37	1.92 (17.05)	−2.81 (21.36)	0.16	0.49 (17.84)	−0.88 (22.68)	1.37	0.84
Technical skills	80.90 (19.10)	75.10 (20.80)	0.09	0.35 (19.90)	−1.32 (23.01)	0.60	−1.62 (18.12)	0.0003 (28.84)	−1.62	0.97
Information	75.50 (20.60)	68.20 (24.6)	0.06	2.87 (20.60)	−2.71 (26.23)	0.20	2.69 (21.34)	−1.66 (21.86)	4.35	0.33
Availability	77.50 (21.20)	73.10 (23.00)	0.23	1.99 (17.46)	−3.53 (26.04)	0.16	0.78 (18.49)	−4.06 (26.00)	4.84	0.37
Satisfaction with Services										
Interpersonal quality/information	76.30 (19.10)	72.90 (21.10)	0.32	4.75 (16.53)	−5.09 (25.94)	0.06	−0.67 (21.46)	−3.26 (19.02)	2.59	0.51
Waiting time	70.20 (23.90)	65.10 (25.60)	0.21	4.17 (22.92)	−1.70 (26.08)	0.15	6.25 (21.81)	−4.60 (25.07)	10.85	0.11
Accessibility	61.80 (26.10)	55.50 (24.80)	0.16	5.83 (27.12)	−1.16 (28.58)	0.11	4.92 (26.50)	−1.60 (24.53)	6.52	0.28
Exchange of information	69.60 (24.00)	64.50 (25.80)	0.23	5.68 (25.21)	−4.07 (29.34)	0.06	3.12 (30.29)	0 (25.75)	3.12	0.33
Comfort	70.60 (23.60)	65.70 (23.40)	0.14	7.05 (24.30)	2.38 (29.18)	0.08	5.01 (21.17)	1.31 (27.84)	3.68	0.42
Overall Satisfaction	77.50 (19.60)	72.42 (22.10)	0.15	5.00 (18.08)	- 0.61 (22.69)	0.16	3.79 (17.81)	−0.74 (23.86)	4.5	0.27

*p*-Value calculated from Mann–Whitney tests. Control; MD: mean difference between intervention vs. control.

**Table 5:** Concomitant treatments.

Concomitant Treatment	TOTAL (N = 200)	Intervention (N = 100)	Control (N = 100)	*p*-Value
	*n* (%)	*n* (%)	*n* (%)	
Painkillers				0.1552
No	32 (17.98)	20 (21.98)	12 (13.79)	
yes	146 (82.02)	71 (78.02)	75 (86.21)	
Missing data	22	9	13	
Antidiarrhea				0.2120
No	160 (93.57)	79 (90.80)	81 (86.43)	
yes	11 (6.43)	8 (9.20)	3 (3.57)	
Missing data	29	13	16	
Antiemetic				0.2325
No	90 (51.14)	51 (55.43)	39 (46.43)	
yes	86 (48.64)	41 (44.57)	45 (53.57)	
Missing data	24	8	16	
Psychotropics				0.8963
No	120 (68.57)	62 (68.13)	58 (69.05)	
yes	55 (31.43)	29 (31.87)	26 (30.95)	
Missing data	25	9	16	
Diet				0.1821
No	54 (31.76)	32 (36.36)	22 (26.83)	
yes	116 (68.24)	56 (63.64)	60 (73.71)	
Missing data	30	12	18	
Antibiotic				0.9258
No	120 (68.57)	62 (68.89)	58 (68.24)	
yes	55 (31.43)	28 (31.11)	27 (31.76)	
Missing data	25	10	15	

## Data Availability

The data presented in this study are available on request from corresponding author.

## Supplementary Materials

The following are available online at https://www.mdpi.com/article/10.3390/cancers13153826/s1, Figure S1: Baseline mean scores from the intervention questionnaires QLQ-C30 and QLQ-N&H35, Table S1: Mixed analyses of EQ-D5-VAS. Satisfaction with waiting times and satisfaction with accessibility.
