# Supplementary material for: Randomized Trial Assessing the Impact of Routine Assessment of Health-Related Quality of Life in Patients with Head and Neck Cancer
Source: Cancers (Basel). 2021 Jul 29;13(15):3826. doi: 10.3390/cancers13153826 (PMC8345055; doi:10.3390/cancers13153826)
Supplement: Supplementary file 1 [file cancers-13-03826-s001.zip › cancers-1288480-supplementary.pdf]

cancers

Supplementary materials

Randomized trial assessing impact of routine assessment of health-related quality of life in patients with head and neck cancer.

Oumar Billa, Franck Bonnetain, Jérôme Chamois, Angeline Ligey, Valérie Ganansia, Georges Noel, Sophie Renard, Sophie Maillard, Magali Quivrin, Noémie vulquin, Pierre Truntzer, Tienhan Sandrine Dabakuyo-Yonli, Philippe Maingon

A

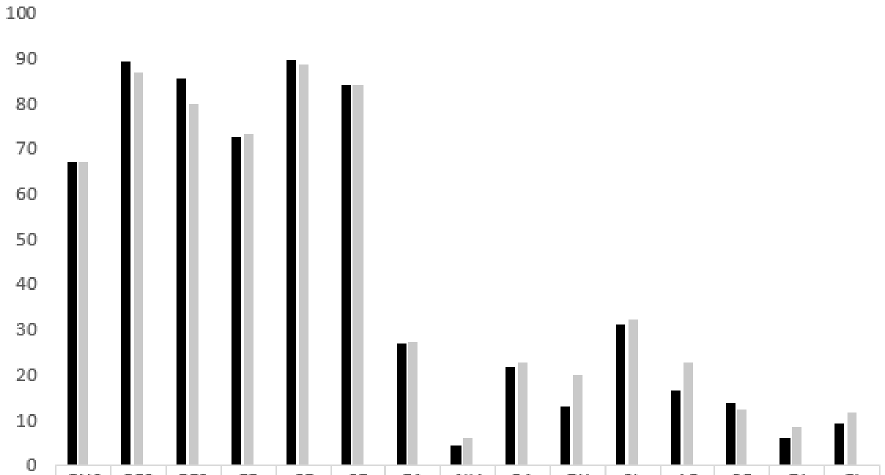

	GHS	PF2	RF2	EF	CF	SF	FA	NV	PA	DY	SL	AP	CO	DI	FI
ARM A	67.02	89.1	85.48	72.7	89.71	83.86	26.88	4.16	21.8	12.96	31.11	16.28	13.47	6.03	9.22
ARM B	67.04	86.71	79.85	73.35	88.64	83.9	27.16	5.86	22.53	19.78	32.23	22.6	12.08	8.52	11.48
p value	0.84	0.44	0.08	0.8	0.98	0.84	0.97	0.88	0.54	0.13	0.77	0.18	0.82	0.19	0.28

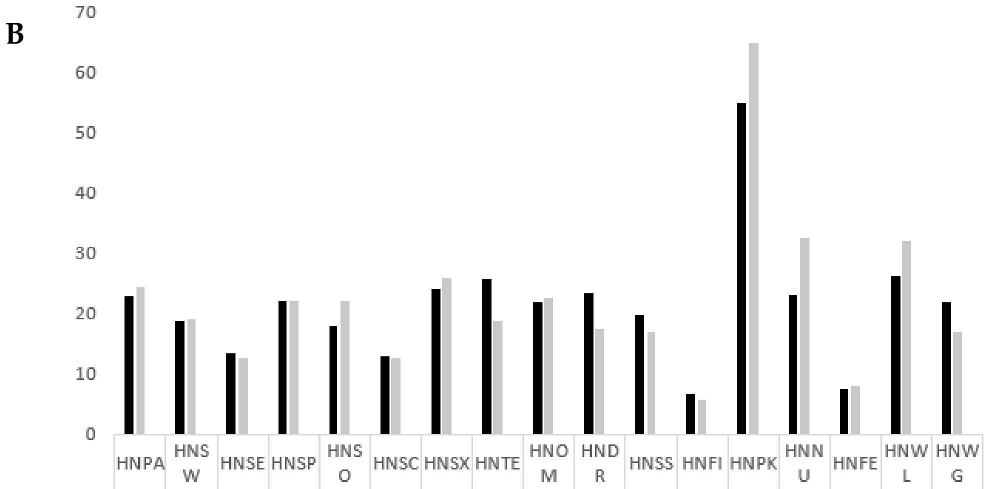

	HNPA	HNSW	HNSE	HNSP	HNSO	HNSC	HNSX	HNTE	HNOM	HNDR	HNSS	HNFI	HNPK	HNNU	HNFE	HNWL	HNWG
ARM A	22.8	18.7	13.3	22.2	17.9	12.8	24.1	25.7	21.8	23.5	19.7	6.8	54.8	23.1	7.5	26.1	21.9
ARM B	24.5	19.1	12.5	22.2	22.2	12.5	25.9	18.7	22.53	17.6	17	5.6	64.8	32.6	7.9	32.2	17
p value	0.51	0.96	0.77	0.62	0.58	0.98	0.86	0.2	0.98	0.37	0.33	0.74	0.17	0.15	0.93	0.37	0.4

Figure S1: Baseline means scores from intervention questionnaires.

(A) Baseline means scores for EORTC-QLQ-C30 by arm. Global health status (GHS), physical (PF2), role (RF2), emotional (EF), cognitive (CF) and social functioning (SF). Fatigue (FA), nausea/vomiting (NV), pain (PA), dyspnea (DY), insomnia (SL), appetite loss (AP), constipation (CO), diarrhea (Di) and financial difficulties (FI) scales.

(B) Baseline means scores for EORTC-QLQ H&N35 by arm. Symptom's scales/ items: pain (HNPA), swallowing (HNSW), senses problems (HNSE), speech problems (HNSP), trouble with social eating (HNSO), trouble with social contact (HNSC), less sexuality (HNSX), teeth (HNTE), opening mouth (HNOM), dry mouth (HNDR), sticky saliva (HNSS), felt ill (HNFI), pain killers (HNPK), nutritional supplements (HNNU), feeding tube (HNFE), weight loss (HNWL) and weight gain (HNWG).

*Arm A=Intervention arm

*Arm B= Control arm

Table S1: mixed analyses of EQD5-VAS. Satisfaction with waiting times and satisfaction with accessibility[¥]

Covariates	Estimate of effects (SE)	95% CI	p
**Global health state (EQ VAS)** [≠]			
Intercept	56.66 (9.90)		<.0001
Follow-up			0.002*
At baseline	Ref.		
At 12 months	3.56 (2.36)	(0.91 to 6.84)	0.01
At 24 months	4.21 (2.62)	(2.85 to 10.47)	0.0007
Study arm (Intervention Vs. control)	-2.67 (3.33)	(-11.35 to 6.01)	0.42
Interaction (follow up x study arm)			0.32*
**Satisfaction with Waiting times**[≠]			
Intercept	67 (10.29)		
Follow-up			0.64*
At baseline	Ref.		
At 12 months	-1.21 (3.43)	(-10.18 to 7.76)	0.72
At 24 months	-1.22 (3.47)	(-10.28 to 7.84)	0.72

**Study arm** (Intervention Vs. control)	3.97 (4.13)	(-6.82 to 14.77)	0.34
**Interaction** (follow up x study arm)	Ref.		
**Satisfaction with access to hospital**≠			
Intercept	62.26 (11.16)		<.0001
Follow-up			0.77*
At baseline	Ref.		
At 12 months	-2.52 (3.91)	(-12.72 to 7.68)	0.52
At 24 months	-1.45 (3.70)	(-11.11 to 8.22)	0.70
Study arm (Intervention Vs. control)	5.17 (4.50)	(-6.57 to 16.92)	0.25
Interaction (follow up x study arm)			0.34*

¥ Each dimension was analyzed as outcome in an individual multivariate model; EQD VAS from Europol; satisfaction with waiting times and satisfaction with access to hospital from EORTC QLQ-SAT32

*Overall effect

P value from linear mixed-model analysis (two-sided).

CI: confidence interval

≠ adjusted on age, alcohol, comorbidity, treatment center, cancer site and cancer stage
